# The effects of intravitreal adalimumab injection on pseudophakic macular edema

**DOI:** 10.1186/s13104-020-05197-w

**Published:** 2020-07-25

**Authors:** Mohsen Farvardin, Ehsan Namvar, Fatemeh Sanie-Jahromi, Mohammad Karim Johari

**Affiliations:** Poostchi Ophthalmology Research Center, Department of Poostchi Ophthalmology, Shiraz University of Medical Sciences, Zand Street, Shiraz, Fars Iran

**Keywords:** Pseudophakic macular edema, Adalimumab, Intravitreal injection

## Abstract

**Objective:**

Pseudophakic macular edema is a frequent complication following cataract surgery. Inflammation is a major etiologic factor in the development of pseudophakic cystoid macular edema. Tumor necrosis factor-alpha has an important role in ocular inflammation. Adalimumab (Humira) is an inhibitor of tumor necrosis factor-alpha that has been approved in the United States. An open-label, uncontrolled, prospective, interventional study of five consecutive patients (5 eyes) with cystoid macular edema who were treated with off-label intravitreal adalimumab at Khalili Hospital was conducted. Slit-lamp examination and optical coherence tomography were done for all patients.

**Results:**

No statistically significant difference was detected between best corrected visual acuity and central macular thickness before and after injection in pseudophakic macular edema. One patient developed uveitis approximately 2 weeks after injection. Based on the results, adalimumab does not appear to be an effective treatment for pseudophakic macular edema, and it may cause uveitis. Caution should be exercised when using this drug.

*Trial registration* Iranian Registry of Clinical Trials IRCT2016100430130N1, 2016.12.03, Retrospectively registered

## Introduction

pseudophakic macular edema is one of the frequent complications of cataract surgery which is a severe postoperative inflammatory response [1, 2]. Inflammation is generally accepted as the main etiologic factor in the development of pseudophakic cystoid macular edema (CME). It has been shown that postsurgical inflammation may increase the permeability of the blood-retinal barrier (BRB) after cataract surgery, resulting in macular edema [1, 3]. CME if left untreated can lead to vision loss [4] and so different therapeutic strategies have been so far introduced for CME. Treatments for CME comprise topical, periocular, intravitreal and systemic corticosteroids [1, 5, 6], systemic and topical nonsteroidal anti-inflammatory drugs (NSAIDs) [1, 7], systemic and topical carbonic anhydrase inhibitors [1], laser therapy [8], pars plana vitrectomy [9], and intravitreal anti-vascular endothelial growth factor (VEGF) agents [10, 11]. Some patients, however, do not respond to the treatments, which represents an unmet need for alternative medicines targeting CME. Tumor necrosis factor-alpha (TNF-α) is a cytokine released from macrophages/monocytes and is known to have an important role in ocular inflammation [12–14]. Recent reports have confirmed the efficiency of TNF-α inhibition in reducing ocular inflammations [14–16]. Adalimumab (Humira) is the newest antibody against TNF- α which has been approved in the United States as an anti-rheumatic drug [17]. It is a human-derived monoclonal antibody and seems to be much safer than its similar drug -infliximab- which is a mouse-human antibody [18].

To the best of the researchers’ knowledge, few studies have been conducted to determine the effects of adalimumab on pseudophakic macular edema; thus, this study was undertaken.

## Main text

### Methods

This prospective, interventional study evaluated five consecutive patients (5 eyes) with CME after uncomplicated cataract surgery with no retinal abnormality (such as vitreomacular traction or diabetic abnormality) who were treated with off-label intravitreal adalimumab at Khalili Hospital. The sample size was determined according to previous studies [15–17]. This study was approved by the ethics committee of Shiraz University of Medical Sciences (IR.SUS.REC.1394.99) and Iranian Registry of Clinical Trials (IRCT2016100430130N1) and patient informed written consent were obtained. The drug and its potential risks were described for all patients. This study adheres to CONSORT guidelines and includes a completed CONSORT checklist as an additional file submitted to the journal. Clinical examination included best corrected visual acuity (BCVA), applanation tonometry, slit-lamp examination, fundus examination, and optical coherence tomography (OCT) and was performed on all patients. Baseline retinal thickness was obtained by OCT. A vial of adalimumab (40 mg/0.8 ml) was mixed with 0.8 ml of balanced salt solution. Then, 0.04 ml (1 mgofadalimumab) of this solution was prepared for each patient. Each eye was prepared with povodine-iodine 5%, the eyelid speculum was inserted, and 1 mg (0.04 ml) of adalimumab was injected at 3.5 mm posterior to the limbus with a 30-gauge needle under topical anesthesia. Afterward, a topical antibiotic was administered for 3 days. Patients were given detailed post-injection instructions and asked to refer to the hospital if any pain or decrease in vision occurred.

Case no. 1 received topical NSAID and topical steroid 2 weeks after uncomplicated cataract surgery for 2 months. Then, adalimumab was injected intravitreally. Case no. 2 received 1.5 months of topical NSAID and a topical steroid after uncomplicated cataract surgery. Thereafter, adalimumab was injected intravitreally. Cases no. 3 and 4 received subtenon triamcinolone after one month of using topical NSAID and steroids. Then, adalimumab was injected intravitreally. Case no. 5 was prescribed topical NSAID and steroids for 1 month followed by subtenon steroid injection. 2 months after uncomplicated cataract surgery, the intravitreal steroid was injected. 3 months later, adalimumab was injected intravitreally.

Slit-lamp biomicroscope and OCT was done for all patients before and after injection. Statistical analysis was performed. The patients’ BCVAs were converted to a logarithm of the minimal angle of resolution (logMAR) scale for analysis. A *p* value < 0.05 was considered to be significant.

### Results

Five eyes from 5 patients (3 men and 2 women) were included in this study. The mean age of the patients was 64.2 years (range = 47–74 years). Mean logarithms of the minimum angle of resolution of BCVA 1 day before, 1 week after, and 4 weeks after injection were 0.9000, 0.8380, and 0.9950, respectively.

The logarithm of the minimum angle of resolution ranged from 0.52 to 1.77. The median logarithms of the minimum angle of resolution before, 1 week after, and 4 weeks after injection were 0.6900, 0.6900, and 0.8450, respectively. (Table 1).

**Table 1:** Demographic characteristics, CMT, and BCVA of study patients

Patient No	Eye	Sex	BCVA1	BCVA2	BCVA3	CMT1	CMT2	CMT3
1	OD	Female	0.52	0.52	0.52	464	505	356
2	OD	Female	0.52	0.52	0.69	567	371	492 (422)
3	OS	Male	1.77	1.77	1.77	968	410	394
4	OD	Male	1.00	0.69	1.00	406	385 (413)	423
5	OD	Female	0.69	0.69	–	512	571	–

*BCVA1* the logarithm of the minimum angle of resolution BCVA 1 day before injection, *BCVA2* the logarithm of the minimum angle of resolution BCVA 1 week after injection; *BCVA3* the logarithm of the minimum angle of resolution BCVA 1 month after injection, *CMT1* central macular thickness 1 day before injection, *CMT2* central macular thickness 1 week after injection, *CMT3* central macular thickness 1 month after injection, *OD* right eye, *OS* left eye

There were no statistically significant differences among BCVA values before, 1 week after, and 4 weeks after injection (*p* value = 0.667, > 0.05).

There were no statistically significant differences among central macular thickness (CMT) values before, 1 week after, and 4 weeks after injection (*p* value = 0.653, > 0.05).

**Fig. 1:**
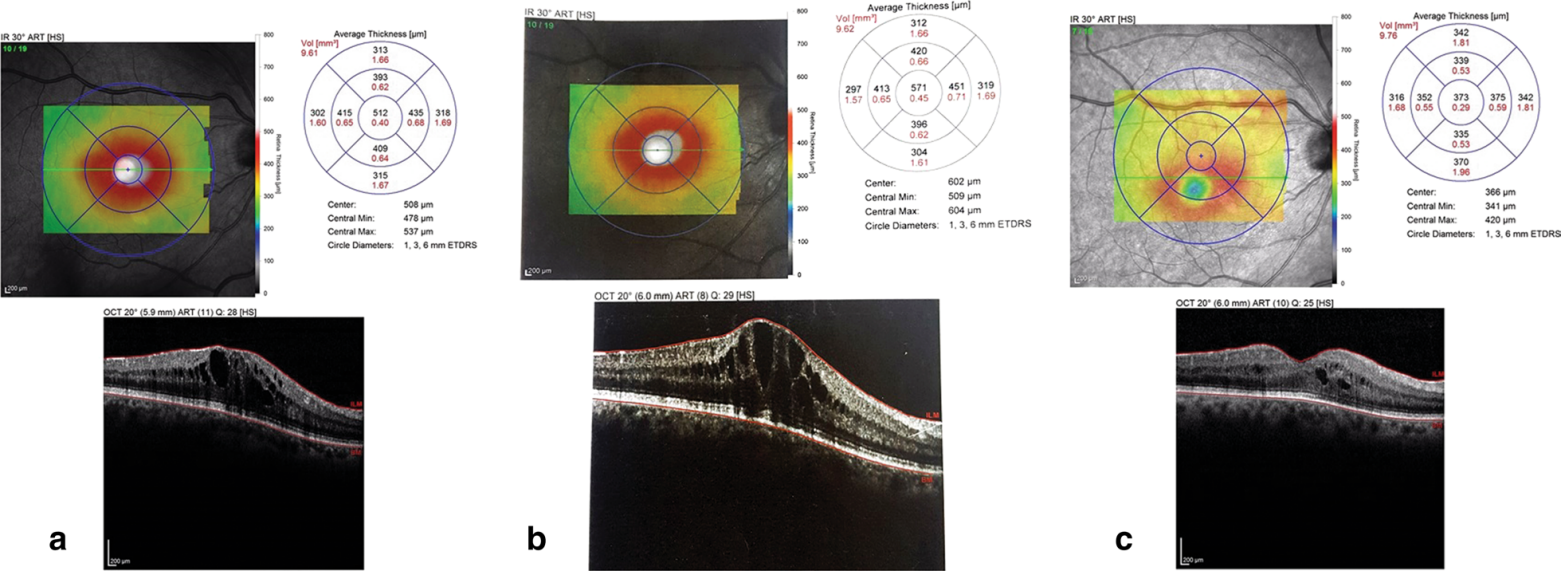
OCT scans of the patient with sterile endophthalmitis (**a**) before injection, (**b**) 1 week after injection, (**c**) after vitrectomy. OCT represents CMT of 512 before injection, 571 1 week after injection that decreased to 373 after vitrectomy

Case 5 developed uveitis approximately 2 weeks after injection. She presented with red eye and ocular pain. Her intraocular pressure (IOP) had risen. Slit-lamp examination showed conjunctival hyperemia, anterior chamber reaction, multiple keratic precipitates, and vitritis. Anterior chamber and vitreous sampling and intravitreal antibiotic injection were done. Smear and culture were negative, but the patient’s condition worsened and visual acuity decreased. Eventually, because of uncontrolled uveitis and increased IOP, a pars plana vitrectomy was done. Thereafter, the uveitis resolved and the IOP became normal. OCT showed a CMT of 373 after vitrectomy and about 2 months after adalimumab injection (Fig. 1).

### Discussion

This study explored the potential for adalimumab to be added to the armamentarium against post-cataract surgery inflammation. Given past evidence for efficacy of the mouse-originated infliximab, it was hypothesized that the humanized TNF-α inhibitor agent adalimumab may have beneficial effects with greater safety. [19–21]. Adalimumab is the most recent anti-TNF-α introduced and approved by the United States. This drug has been widely studied for the treatment of uveitis [17]. Although promising results were achieved on the anti-inflammatory effect of adalimumab in uveitis [17] little is known about the effect of adalimumab in cataract induced CME. The present study was designed to evaluate the effect of adalimumab in patients with pseudophakic macular edema. In our small patient cohort, beneficial outcomes were not achieved. There are several reports on the safety and efficacy of adalimumab in animal and experimental models. In rabbit (vitreous volume = 1.5 ml), the intravitreal administration of 0.50 mg adalimumab is safe, but 1.0 mg results in inflammation and retinal necrosis [19]. Androudi et al. [20] demonstrated that 1.0 mg of adalimumab was not associated with adverse effects. Similarly, Manzano et al. [19] reported that the mean concentrations of adalimumab after the injection of 0.25, 0.50, and 1.0 mg were 0.17, 0.33, and 0.67 mg/ml, respectively, which are comparable to the injection of 0.75, 1.5, and 3.0 mg adalimumab in the human eye according to the vitreous volume. Lihteh Wu et al. [16] demonstrated that intravitreal infliximab causes both anatomical and functional improvement in refractory pseudophakic CME, though no benefit in terms of vision gain and retinal thickness reduction were noted in our 5 patients with pseudophakic CME.

In another study, Lihteh Wu et al. [21] reported no benefit from intravitreal adalimumab and infliximab injections in patients with refractory diabetic macular edema. It seems that intravitreal injections of infliximab may lead to a severe intraocular inflammatory reaction. In the present study, one patient developed uveitis about 2 weeks after adalimumab injection which shows the risk of intravitreal injection of this drug.

Sofia Androudi et al. [20] reported no improvement in BCVA or central retinal thickness (CRT) reduction in patients with chronic uveitic macular edema treated with intravitreal adalimumab. In 2012, Farvardin et al. [22] reported BCVA improvement and decreased CMT in patients with chronic noninfectious uveitis treated with intravitreal infliximab, but its effect was temporary.

In the current study we also showed that adalimumab can lead to a transient decrease in CMT but there was no statistically significant difference in CMT values before injection, 1 week after injection, and 4 weeks after injection; nor was there any statistically significant difference in BCVA before injection, 1 week after injection, and 4 weeks after injection. Therefore, no beneficial effect for vision nor macular thickness was appreciated among our patients with pseudophakic macular edema treated with adalimumab. Additionally, inflammation-related safety concerns may also exist for this agent. Further study is needed to evaluate the efficacy and safety of intravitreal adalimumab. According to the available data, this drug should be used with greater caution.

## Limitations

Of the limitations of our study were the small sample size and the lack of a control group (including patients receiving intravitreal steroids). So, further study is suggested to evaluate the efficacy and safety of intravitreal adalimumab.

## Data Availability

Not applicable.

## Abbreviations

BCVA: Best corrected visual acuity
BRB: Blood-retinal barrier
CME: Cystoid macular edema
CMT: Central macular thickness
CRT: Central retinal thickness
IOP: Intra-ocular pressure
NSAIDs: Non-steroidal anti-inflammatory drugs
OCT: Optical coherence tomography
TNF-α: Tumor necrosis factor-alpha
VEGF: Vascular endothelial growth factor
